# Notes and Notices

**Published:** 1891-06

**Authors:** 


### NOTES AND NOTICES.

The Glen Mary Home, a private homœopathic asylum, has been opened at Owego, Tioga County, N. Y., by Dr. A. J. Givens, formerly of Middletown and Wesborough Insane Asylums.

Dr. Frank Kraft, Professor of Materia Medica, etc., in the Cleveland Homœopathic Hospital College, has removed to 1905 Euclid Avenue, Cleveland, Ohio, where he will continue the practice of his profession, as well as his excellent lectures on materia medica. His specialty is materia medica, he is therefore particularly suitable as consulting physician in difficult cases.

The Rhode Island Homœopathic Society, at its April meeting, unanimously voted that the *American Institute of Homœopathy* be invited to hold the session of 1892 within the boundaries of that State. It is understood, accidents excepted, that the particular place will be the "Ocean House," Newport, and the time the fourth week in June.

Removals.—Dr. R. S. Kirkpatrick, from Harlan, Iowa, to 609 East Locust Street, Des Moines, Iowa. Dr. C. H. Krause has located at 2808 Taylor Avenue, St. Louis. Dr. O. F. Hill, from Epworth, Iowa, to Englewood, Ill. Dr. W. C. McDowell, from Mt. Pleasant to Morning Side Sanitarium, Sioux City, Iowa. Dr. George W. Dunn, from Atlanta, Illinois, to Tiffin, Ohio. Dr. F. M. Leitch, from Lerna to Charleston, Illinois. Dr. Charles F. Hitchcock, from St. Louis to Warner's, Onondaga County, N. Y. Dr. J. B. Sullivan, from 210 Penn Avenue to Butler and 41st Streets, Pittsburgh, Pa. Dr. F. Keller, from Trinidad, Colorado, to Moscow, Idaho. Dr. J. W. Thomson, from 114 West 16th Street, to 248 West 14th Street, New York City. Dr. M. Florence Taft, from Middletown to Waterbury, Conn. Dr. Stuart Close, from 182 Hart Street, to 641 Willoughby Avenue, Brooklyn. Dr. M. R. Jamison, from Connellsville to 165 43d Street, Pittsburgh, Pa. Dr. C. C. Howard, from 49 East 59th Street to 64 West 51st Street, New York. Dr. Thomas M. Stewart, removed to 104 West Eighth Street, Cincinnati, Ohio. Dr. F. R. Schmucher, removed to 228 North 5th Street, Reading, Pa.

